# Reactive Oxygen Species Released from Hypoxic Hepatocytes Regulates MMP-2 Expression in Hepatic Stellate Cells

**DOI:** 10.3390/ijms12042434

**Published:** 2011-04-07

**Authors:** Jing Li, Renhua Fan, Susu Zhao, Leilei Liu, Shanshan Guo, Nan Wu, Wandong Zhang, Pingsheng Chen

**Affiliations:** 1 Department of Pathology, School of Medicine, Southeast University, Nanjing 210009, China; E-Mails: 8218lijing@sina.com (J.L.); 493069029@qq.com (R.F.); lilyy778@163.com (L.L.); 973573379@qq.com (N.W.); 153777836@qq.com (S.G.); 2 Department of Pathology, Jiangsu Province Hospital of TCM, Nanjing 210029, China; E-Mail: susu001135@163.com; 3 Institute for Biological Sciences, National Research Council of Canada, Ottawa, Ontario, K1A 0R6, Canada; E-Mail: wdzhang@gmail.com

**Keywords:** hypoxia, hepatocyte, hepatic stellate cells, liver fibrosis, reactive oxygen species

## Abstract

Hypoxia is a common environmental stress factor and is associated with fibrogenesis. Matrix metalloproteinase-2 (MMP-2), produced by hepatic stellate cells (HSCs), plays an important role in liver fibrogenesis. However, inconsistent results have been reported on the impact of hypoxia on MMP-2 expression and activity in HSCs. We speculated that cell–cell interaction is involved in the regulation of MMP-2 expression and activity at low oxygen level *in vivo*. Therefore, in this report we investigated the mechanism by which hypoxic hepatocytes regulates MMP-2 expression in HSCs. Our results showed that the conditioned medium from hypoxia-treated rat hepatocytes strongly induced the expression of MMP-2 mRNA and protein in rat HSC-T6 cells. Reduced glutathione neutralized ROS released from hypoxic hepatocytes, leading to reduced MMP-2 expression in HSC-T6 cells. In addition, phospho-IκB-α protein level was increased in HSC-T6 cells treated with hypoxia conditioned medium, and NF-κB signaling inhibitor inhibited MMP-2 expression in HSC-T6 cells. Taken together, our data suggest that ROS is an important factor released by hypoxic hepatocytes to regulate MMP-2 expression in HSCs, and NF-κB signaling is crucially involved in ROS-induced MMP-2 expression in HSCs. Our findings suggest that strategies aimed at antagonizing the generation of ROS in hypoxic hepatocytes and inhibiting NF-κB signaling in HSCs may represent novel therapeutic options for liver fibrosis.

## 1. Introduction

It is estimated that over 100 million people suffer from liver fibrosis in the world. As the main complication of chronic liver damage, liver fibrosis is a wound healing process characterized by the accumulation of extracellular matrix (ECM) proteins in the liver. Although liver fibrosis is caused by a variety of etiologic agents, including chronic viral hepatitis, alcohol toxicity, autoimmune disease, and hereditary metabolic disorders, it is now generally accepted that a central pathologic mechanism underlying liver fibrosis is the generation and proliferation of smooth muscle α-actin (α-SMA)-positive myofibroblasts of periportal and perisinusoidal origin that arise as a consequence of the activation of hepatic stellate cells (HSCs) [1–3,4]. HSCs play a critical role in the excessive production and secretion of ECM, resulting in the generation of fibrous tissue and scar formation [5]. The activation of HSCs is considered to be the key factor responsible for liver fibrosis [1]. In addition to cytokines and other soluble factors released by Kupffer cells, inflammatory cells and damaged hepatocytes, changes of ECM composition have been suggested to be implicated in the activation of HSCs [6].

Enzymes known to degrade type IV collagen include matrix metalloproteinase-2 (MMP-2) and matrix metalloproteinase-9 (MMP-9), both of which are members of zinc-dependent matrix metalloproteinase family. MMP-2 is involved in ECM remodeling during tissue and organ development, wound repair, tumor invasion and metastasis. It has been shown that activated HSCs proliferate quickly and produce a large amount of ECM and MMP-2 [7,8]. Therefore, MMP-2 and HSCs activation are interdependent. During the progression of liver fibrosis, deposition of type IV collagen increases significantly, which might be due to the inability of MMP-2 to degrade type IV collagen. If the expression and activity of MMP-2 were interfered at different stages, the progression of liver fibrosis may be changed. Thus, further characterization of HSCs is crucial for the understanding of the pathogenesis of hepatic fibrosis.

Many factors have been shown to affect MMP-2 expression and activity in HSCs, such as reactive oxygen species (ROS) generated during oxidative stress, transforming growth factor β1 (TGF-β1), p53, AP-1, membrane-type matrix metalloproteinase (MT1-MMP), tissue inhibitor of matrix metalloproteinase-2 (TIMP-2), plasmin, thrombin, type I collagen, Zn^2+^, reversion-inducing-cysteine-rich protein with Kazal motifs (RECK), and the interaction between hepatocytes and hepatic stellate cells [9–11]. Oxygen is necessary for cellular metabolic process and acts as a modulator of gene expression [12]. One of the most prominently pathological significance of hypoxic mammals is an increase in ECM synthesis. The steady-state concentration of the collagens depends on both the rate of their synthesis and their degradation. Hypoxia is involved in the pathogenic process of liver fibrosis and may activate HSCs [13–15]. In addition, our previous studies have shown that hypoxia stimulated MMP-2 synthesis in HSCs *in vitro*, and the expression of hypoxia inducible factor-1α was increased in hepatocytes in the rat liver fibrosis tissues [16–18]. However, it is unknown whether and how hepatocytes modulate MMP-2 expression in HSCs under hypoxic conditions. Therefore, in this study we investigated the mechanism by which hypoxic hepatocytes regulates MMP-2 expression in HSCs. Our results suggest that ROS is an important factor released by hypoxic hepatocyte to regulate MMP-2 expression in HSCs and NF-κB signaling is crucially involved in these processes.

## 2. Materials and Methods

### 2.1. Cell Culture and Preparation of Conditioned Media

Rat hepatocyte BRL-3A cells and hepatic stellate cell HSC-T6 cells were obtained from Type Culture Collection of Chinese Academy of Sciences and cultured in Dulbecco’s modified Eagle medium (DMEM, Invitrogen, USA) containing 10% fetal calf serum (FBS, Gibco, USA) and 100 units/mL penicillin/streptomycin at 37 °C in a humidified atmosphere with 5% CO_2_. Cells were plated in 6-well plates at a density of 2 × 10^5^ cells/well. At 80% confluence, BRL-3A cells were treated with hypoxia. The cells were replaced with serum-free DMEM and then incubated in the chambers flushed with air mixture containing 1% O_2_, 5% CO_2_ and 94% N_2_ for 30 min. The ultimate oxygen tension was 5%. The chambers were sealed and placed at 37 °C and incubated for 12 h. Controls included parallel cultures in which cells were exposed to ambient air (e.g., normaxia/21% oxygen tension). The supernatant was collected from the cultured cells at 12 h and passed through a 0.22 μm filter. The filtrate was defined as hypoxia conditioned medium and normoxia conditioned medium. The conditioned medium was used immediately for following experiments.

To induce HSC-T6 cell differentiation and MMP-2 expression by hypoxia conditioned medium, HSC-T6 cells were grown to 80% confluence and then washed twice with PBS. Then HSC-T6 cells were cultured in hypoxia conditioned medium and collected after 6 h, 12 h and 24 h, respectively. As controls, HSC-T6 cells were cultured in DMEM medium or normoxia conditioned medium. All the cells were cultured in a humidified atmosphere of 5% CO_2_ and 95% air at 37 °C.

### 2.2. Real-Time RT-PCR

Total RNA was extracted from HSC-T6 cells with Trizol reagent (Invitrogen, USA) following the manufacturer’s instructions. Total RNA (1 μg) was used for cDNA synthesis using PrimeScript RT reagent kit (TaKaRa, Dalian, China). Aliquot of diluted first-strand cDNA was amplified with a Real-Time PCR Detection System (ABI7300, USA) using SYBR PrimeScript RT-PCR Kit according to the manufacturer’s instructions. Glyceraldehyde 3-phosphate dehydrogenase (GAPDH) was used as an internal control. The following primers were used: MMP-2, 5-AGGGCACCTCCTACAACAGC-3 and 5-CAGTGGACATAGCGGTCTCG-3 (126 bp) [19]; GAPDH, 5-GAAGGGCTCATGACCACAGT-3 and 5-GGATGCAGGGATGATGTTCT-3 (117 bp) [20]. RT-PCR was performed three times in triplicate. The relative mRNA level of MMP-2 was compared to that of GAPDH and was calculated by the 2^−^^ΔΔ^^Ct^ method. Each Ct value used for these calculations was the mean of the triplicate for each reaction.

### 2.3. Western Blot

HSC-T6 cells were lysed with RIPA lysis buffer (Beyotime, China). After centrifugation, the lysates were determined by the Bradford assay (Beyotime, China) for protein concentration. Total protein for each sample (50 μg) was fractionated on 10% SDS-PAGE gel and transferred to polyvinylidene difluoride (PVDF) membrane. The membrane was blocked with 5% skimmed milk at room temperature for 1 h and incubated with the rabbit polyclonal anti-MMP-2 antibody (Bioworld, China, 1:500 dilution), phospho-IκB-α antibody (Bioworld, China, 1:500 dilution), IκB-α antibody (Bioworld, China, 1:500 dilution), or mouse monoclonal anti-β-actin antibody (Santa Cruz, USA, 1:1000 dilution) at 4 °C overnight. After washing, the membrane was incubated with horseradish peroxidase-conjugated anti-goat or anti-mouse antibody (Santa Cruz, USA, 1:10,000 dilution) at room temperature for 1 h. After washing, the bound antibody was visualized with Super ECL Detection Reagent (Applygen, China).

### 2.4. Gelatin Zymography for MMP-2

MMP-2 activity was determined by gelatin zymography. Supernatants were collected from HSC-T6 cells and centrifuged. Protein content of the supernatants was determined by the Bradford assay. Total protein for each sample (15 μg) was fractionated on 10% SDS-PAGE gel containing 0.1% gelatin under non-reducing conditions. Gelatin zymography was performed using a MMP Zymography assay kit (Applygen Technologies Inc.) according to the manufacturer’s instructions. Gelatinolytic bands were observed as clear zones against the blue background and the intensity of the bands was estimated using the ScnImage Software.

### 2.5. Quantitative Assay for Reactive Oxygen Species

BRL-3A cells were cultured and treated with hypoxia as previously described in 96-well plates. The final oxygen tensions in the chambers were 5% and 10%, respectively. Controls included parallel cultures in which cells were exposed to normoxia (21% oxygen tension). 12 h later, the supernatants were collected and put in another 96-well cell culture plates. The ROS in the supernatants was measured by ROS assay kit (Genmed, China). Briefly, the samples were incubated with 10 μL 3,3′,5,5′ tetramethylbenzidine (TMB Substrate) at 37 °C for 1 h. This would result in blue color development proportional to the amount of ROS and the level of ROS in each sample was measured with the spectrophotometer at a wavelength of 650 nm.

### 2.6. Treatment of BRL-3A Cells with Reduced Glutathione

BRL-3A cells were cultured in 6-well plates. At 80% confluency, the medium was changed to serum-free DMEM medium containing reduced glutathione (0, 0.5, 2.5, 10 mmol/L). After 30 min, the plates were incubated in a chamber flushed with air mixture containing 1% O_2_, 5% CO_2_ and 94% N_2_ for 30 min. The chambers were sealed and placed at 37 °C and incubated for 12 h. The level of ROS in each sample was measured according to the method above. Meanwhile, the supernatants of BRL-3A were collected from the cells and passed through a 0.22 μm filter. The filtrate was designated as ROS-reduced-hepatocyte-conditioned medium and stored at −20 °C for later use. The ROS-reduced-hepatocyte-conditioned medium was used to culture HSC-T6 cell as previously described for 6 h, 12 h, and 24 h. Then the supernatants were collected, and total protein and RNA were extracted from HSC-T6 cells.

### 2.7. Statistical Analysis

Experiments were repeated at least three times. The results were presented as means ± SEM. Comparison of multiple parameters was performed with one-way ANOVA test (SPSS 11.5), followed by Tukey-Kramer’s *post hoc* test. *p* < 0.05 was considered as statistically significant.

## 3. Results

### 3.1. Hepatocyte Conditioned Medium Upregulates MMP-2 Expression in HSCs

**Figure 1.**
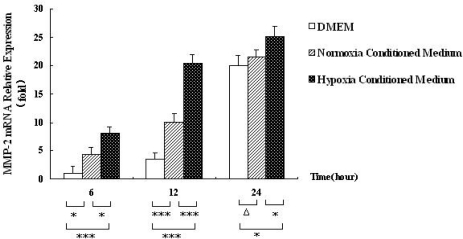
Hepatocyte conditioned medium upregulates MMP-2 mRNA expression in HSCs. Relative MMP-2 mRNA level was determined by Real-time RT-PCR in HSC-T6 cells treated with hepatocyte conditioned medium, serum-free DMEM medium and hepatocyte-conditioned control medium (normoxia conditioned medium) for 6, 12 and 24 h. Data were expressed as means ± SEM from 3 independent experiemnts. * *p* < 0.05, ** *p* < 0.01, *** *p* < 0.001, and Δ*p* > 0.05.

**Figure 2.**
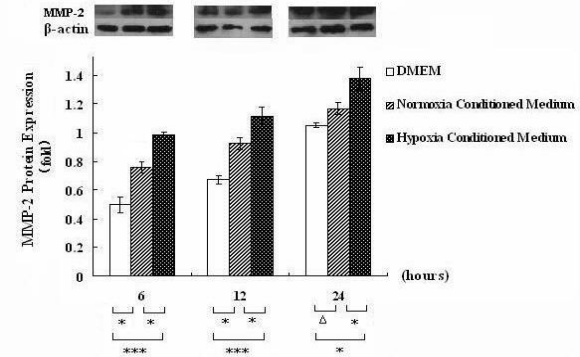
Hepatocyte conditioned medium upregulates MMP-2 protein expression in HSCs. Western blot analysis showing MMP-2 protein level in HSC-T6 cells cultured in hepatocyte conditioned medium, serum-free DMEM medium and hepatocyte-conditioned control medium (normoxia conditioned medium) for 6, 12 and 24 h. The specificity of MMP-2 antibody was demonstrated by the detection of MMP2 as a 72 KD protein. Shown were representative blots from three independent experiments with similar results. Data were expressed as means ± SEM from 3 independent experiments. * *p* < 0.05, and Δ*p* > 0.05.

Our prior study has shown that hypoxia could regulate the expression of MMP-2 mRNA in HSC-T6 cell [16,17,21]. To investigate whether hypoxic hepatocytes affect the expression of MMP-2 mRNA in HSC-T6 cells, we measured MMP-2 mRNA level in rat HSC-T6 cells cultured in hepatocyte-conditioned medium for 6, 12 and 24 h, respectively. As shown in Figure 1, real-time RT-PCR analysis showed that MMP-2 mRNA expression was increased in HSC-T6 cells treated with hepatocyte-conditioned medium. Time factor analysis by one-way ANOVA showed that MMP-2 mRNA level in HSC-T6 cells treated with the conditioned medium was significantly higher than that in the controls (*p* < 0.001). These results indicate that hepatocyte conditioned medium upregulates MMP-2 expression in HSCs. To provide further evidence for this, we performed Western blot analysis to examine MMP2 expression at the protein level in HSC-T6 cells cultured with hepatocyte-conditioned medium at 6, 12 and 24 h, respectively. The results showed that MMP-2 protein was induced in HSC-T6 cells treated with the hepatocyte-conditioned medium. The protein level of MMP-2 at 12 h was higher than that at 6 h and 24 h (Figure 2). Taken together, these data suggest that MMP-2 expression in HSC-T6 cells exhibited a slow response to hepatocyte-conditioned medium.

**Figure 3.**
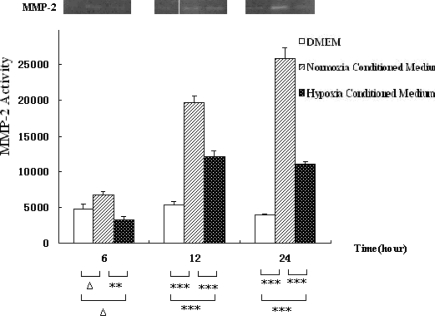
MMP-2 activity measured by gelatin zymography in HSC-T6 cells cultured with hepatocyte conditioned medium (hepatocyte conditioned culture), serum-free DMEM medium, and hepatocyte-conditioned control medium at 6, 12 and 24 h. Typical results were shown from independent experiments performed at least three times. Data were expressed as means ±SEM. * *p* < 0.05, ** *p* < 0.01, *** *p* < 0.001 and Δ*p* > 0.05.

To find out whether anoxic hepatocytes affect the activity of MMP-2 in HSC-T6 cells, gelatin zymography was conducted. The results showed that MMP-2 activity was inhibited by hepatocyte-conditioned medium. The effect within 24 h was more intensive. Time factor analysis by one-way ANOVA test showed that MMP-2 activity in HSC-T6 cells in hepatocyte-conditioned medium and in the controls had significant differences (*p* = 0.024). Group factor analysis (*p* < 0.001) was shown in Figure 3, indicating that hepatocyte-conditioned medium inhibits MMP-2 activity in rat HSCs.

### 3.2. Reduced Glutathione Antagonizes the Generation of ROS in the Supernatants of Hepatocytes

**Figure 4.**
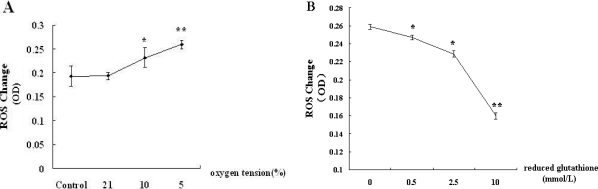
GSH antagonizes the generation of ROS in the supernatants of hepatocytes. (**A**) BRL-3A cells were treated with different levels of oxygen (5% O_2_, 10% O_2_ and 21% O_2_) for 12 h. ROS in the supernatants was quantified using a colorimetric assay. Data were expressed as means ± SEM from 3 independent experiments. * *p* < 0.05, ** *p* < 0.01 *vs.* Control; (**B**) BRL-3A cells were treated with GSH (0, 0.5, 2.5, 10 mmol/L) and then exposed to 5% oxygen for 12 h. The ROS level in the hypoxia conditioned medium was analyzed. Data were expressed as means ± SEM from 3 independent experiments. * *p* < 0.05, *** *p* < 0.001 *vs.* group treated with 0 mmol/L GSH.

Because ROS could regulate MMP-2 expression, we determined the level of ROS in the supernatant of hepatocytes treated with hypoxia. BRL-3A cells were treated with different levels of oxygen (5% O_2_, 10% O_2_, and 21% O_2_) for 12 h and supernatants were collected for quantitative colorimetric assay. The results showed that ROS level gradually increased with the decrease of oxygen tension (Figure 4A). To investigate whether reduced glutathione (GSH) could eliminate the release of ROS to the supernatant of hepatocytes treated with hypoxia, we treated BRL-3A cells with GSH (0, 0.5, 2.5, 10 mmol/L), and examined ROS level in the conditioned medium. The results showed that the level of ROS in hypoxia conditioned medium decreased gradually with the increase of reduced glutathione (Figure 4B).

### 3.3. ROS Generated by Hypoxic Hepatocyte Contributes to the Downregulation of MMP-2 Expression and Activity in HSCs

**Figure 5.**
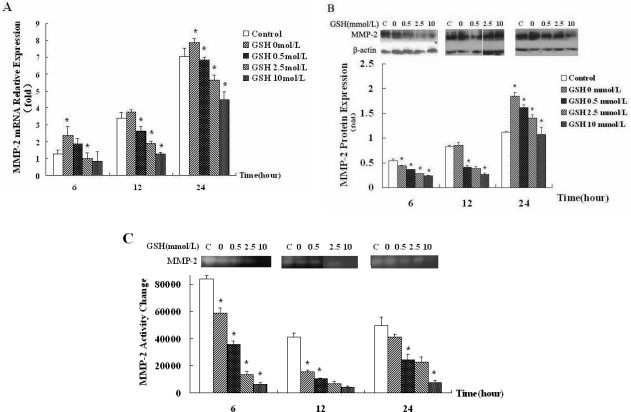
ROS-neutralized hepatocyte conditioned medium downregulates MMP-2 expression and activity in HSCs. BRL-3A cells were treated with reduced glutathione (0, 0.5, 2.5, 10 mmol/L) and exposed to 5% oxygen for 12 h. Then the supernatant was collected and HSC-T6 cells were cultured with ROS-neutralized-hepatocyte-conditioned medium for 6, 12 and 24 h. As a control, HSC-T6 cells were cultured in serum-free DMEM medium. (**A**) relative MMP-2 mRNA level in HSC-T6 cells were determined by Real-time RT-PCR. Data were expressed as means ± SEM from 3 independent experiments. * *p* < 0.05 *vs.* the former group; (**B**) MMP-2 protein level in HSC-T6 cells was detected by Western blot. Shown were representative blots from three independent experiments with similar results; (**C**) MMP-2 activity measured by gelatin zymography.

To confirm that ROS generated by hypoxic hepatocyte is one of the main factors that contribute to increased expression of MMP-2 in HSCs, we examined MMP-2 expression at both mRNA and protein levels in HSC-T6 cells cultured in ROS-neutralized-hepatocyte conditioned medium. Real-time RT-PCR analysis showed that with the increase of GSH concentration, MMP-2 mRNA expression in HSC-T6 cells was significantly inhibited (Figure 5A, *p* < 0.001). In addition, Western blot analysis showed that MMP-2 protein expression in HSC-T6 cells was reduced corresponding to increased GSH concentration (Figure 5B, *p* < 0.001). The result of gelatin zymography (Figure 5C) was consistent with Real-time RT-PCR and Western blot (*p* < 0.001).

### 3.4. Hepatocyte Conditioned Medium Upregulates MMP-2 Expression in HSCs via NF-κB Signaling

**Figure 6.**
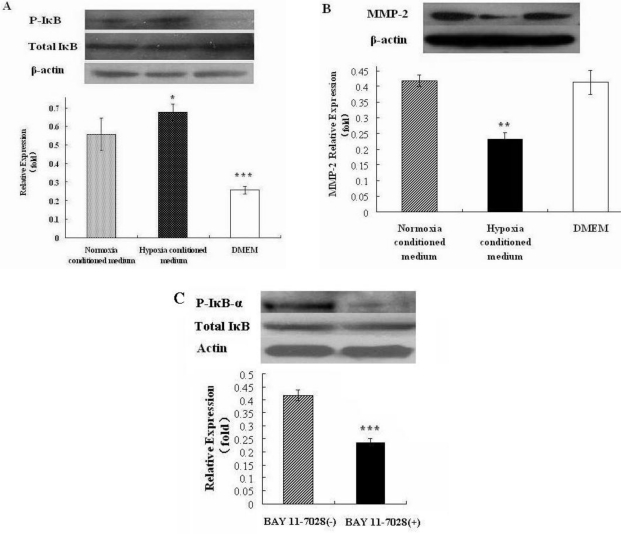
NF-κB signaling mediates ROS-induced MMP-2 expression in HSCs. (**A**) Western blot analysis showing MMP-2 and phosphor-IκB-α levels in HSC-T6 cells cultured in hepatocyte conditioned medium, serum-free DMEM medium and hepatocyte-conditioned control medium (normoxia conditioned medium). Data were expressed as means ± SEM from 3 independent experiments. * *p* < 0.05, Δ*p* > 0.05; (**B**) HSC-T6 cells were treated by BAY 11-7082 (100 μM) or vehicle control and MMP-2 protein level was detected by Western blot. Data were expressed as means ± SEM from 3 independent experiments. * *p* < 0.05 *vs.* vehicle control; (**C**) HSC-T6 cells were treated by BAY 11-7082 (100 μM) or vehicle control, total and phosphor IκB-α levels were detected by Western blot. Data were expressed as means ± SEM from 3 independent experiments. *** *p* < 0.01 *vs.* vehicle control.

To investigate whether ROS in hepatocyte conditioned medium modulates MMP2 expression via NF-κB signaling, we measured the level of phospho-IκB-α protein in HSC-T6 cells cultured with serum-free DMEM, normoxia conditioned medium, hypoxia conditioned medium, respectively, for 12 h. Western blot analysis showed that phospho-IκB-α protein level in HSC-T6 cells treated with the hypoxia conditioned medium was increased compared to that in cells cultured in normoxia conditioned medium (Figure 6A, *p* < 0.05). BAY 11-7082 is a commonly used NF-κB inhibitor. When we treated HSC-T6 cells with BAY 11-7082, we found that MMP2 expression in HSC-T6 cells was significantly inhibited compared to cells treated with vehicle control (Figure 6B, *p* < 0.05). As expected, BAY 11-7082 inhibited the activation of NF-κB in HSC-T6 cells (Figure 6C). Collectively, these data suggest that NF-κB signaling mediates ROS-induced MMP-2 expression in HSCs.

## 4. Discussion

Hypoxia is crucially involved in acute and chronic liver injury. As a repair process in response to a variety of chronic injury stimuli, the progression of liver fibrosis is accompanied by hypoxia. Following a fibrogenic stimulus, HSCs are activated which then synthesize and deposit a large amount of ECM in the liver [22–24]. The activation of HSCs is a key event of fibrogenesis [4,7]. We have found that hypoxia induced the expression of MMP-2 at both mRNA and protein levels, but inhibited its activity in the *in vitro* assay [16–18]. *In vivo* experiment suggested that at the beginning of liver fibrosis, MMP-2 expression and activity were decreased, and fewer HSCs were activated. However, during the development of fibrosis, MMP-2 activity was increased, which degrades collagen IV [17,25–27]. Given these inconsistent results between *in vivo* and *in vitro* studies, we believe that cell–cell interaction is involved in the regulation of MMP-2 expression and activity at low oxygen level *in vivo*. Therefore, in this report we investigated the mechanism by which hypoxic hepatocytes regulates MMP-2 expression in HSCs.

First, we examined whether hypoxic hepatocytes affect MMP-2 expression in HSC-T6 cells. Real-time RT-PCR analysis showed that hypoxic hepatocytes may secrete some factor(s) to affect MMP-2 expression at mRNA level in HSC-T6 cells. Consistent with RT-PCR analysis, Western blot analysis further showed that hepatocyte-conditioned medium induced the expression of MMP-2 in HSC-6T cells at protein level. Taken together, these data indicate that hypoxic hepatocytes release some factor(s) to upregulate the expression of MMP-2 in HSC-T6 cells.

Next, a serial of experiments was conducted to identify the factor(s) released from hypoxic hepatocytes responsible for the upregulation of MMP-2 expression in HSCs. It is known that ROS serves as a signal molecule that regulates many important cellular events, such as transcription factor activation, gene expression, cell differentiation and proliferation. ROS is also involved in the regulation of MMP-2 expression and activation, mainly through Ras and mitogen activated protein kinase (MAPK) signaling cascades [21,28]. Therefore, we detected ROS level in the supernatant of BRL-3A cells treated with different levels of oxygen and found that with the decrease of oxygen tension, ROS was gradually increased. Reduced glutathione (GSH) is a non-protein antioxidant and can protect cells from ROS damage. After treatment with GSH, we detected ROS in the supernatants of hypoxia conditioned medium to determine whether ROS can be scavenged by reduced glutathione. The results evidently demonstrated that as the concentration of GSH increased, the level of ROS in the supernatants of hypoxia conditioned medium was gradually decreased, proving that ROS generated in hepatocytes was scavenged efficiently by reduced glutathione.

To provide further evidence that ROS generated in hepatocytes contributes to the upregulation of MMP-2 expression in HSC-T6. Last, we characterized the potential mechanism by which ROS regulates MMP-2 expression in HSCs. By probing phospho-IκB-α protein level as an indication of the activation of NF-κB signaling, we found that hypoxia conditioned medium induced increased phospho-IκB-α protein level in HSC-T6 cells, corresponding to increased MMP-2 protein level. Furthermore, inhibition of NF-κB signaling by specific inhibitor BAY 11-7082 led to reduced MMP2 protein level in HSC-T6 cells. Based on these data we speculate that NF-κB signaling is an important, although not exclusive, signaling pathway that mediates ROS-induced MMP-2 expression in HSCs.

Gelatin zymography analysis (Figure 5C) showed that hypoxic hepatocytes released ROS to persistently inhibit the activity of MMP-2 in HSC-T6 cell within 24 h. This resutl was consistent with that of Real-time RT-PCR and Western blot. However, in contrast to the result showed in Figure 3, we found that MMP-2 activity was inhibited by hepatocyte-conditioned medium. These results suggest that ROS may be the main factor released by anoxic hepatocyte to regulate MMP-2 mRNA and protein expression, but may not be the main factor that regulates MMP-2 activity.

MMP-2 is secreted as a latent form of zymogen and can be activated when the pro-domain is cleaved and bound to TIMP-2 and MT1-MMP. Many factors are involved in regulating MMP-2 activity. As the main inhibitor, tissue inhibitor of metalloproteinase (TIMP) is the most important regulator of MMP-2 activity. It is possible that the factor released by anoxic hepatocyte regulates MMP-2 activity by means of disturbing the balance between MMP-2 and its endogenous inhibitors TIMPs [29]. As a novel membrane-anchored matrix metalloproteinase (MMP) inhibitor, reversion-inducing cysteine-rich protein with Kazal motifs (RECK) also plays a role in the regulation of MMP activity. RECK inhibits MMP-2, MMP-9, and membrane type-1 MMP (MMP-14) secretion and activity [30]. MMP-2 is Zn-dependent enzyme. Therefore, Zn^2+^ is necessary for MMP-2 activity [31]. In addition, MMP-2 is also directly activated by oxidizing the sulphydril bond between a cysteine residue of the prodomain and the Zn^2+^ catalytic center, resulting in partial enzyme activation followed by an intramolecular cleavage of the propeptide [32]. In our future study we will identify the factors generated by anoxic hepatocytes that inhibit MMP2 activity.

MMP-2 has been shown to promote the formation and development of liver fibrosis, mainly through the regulation of the activation, proliferation, and migration of HSCs. *In vitro* studies found that MMP-2 could degrade type IV collagen-rich matrix around the HSCs, which could activate HSCs. The activated HSCs proliferate actively, produce large amounts of extracellular matrix, and are main source of MMP-2 in the liver [33]. In addition, oxidative stress induced by some pathogenic factors promoted the proliferation and migration of HSCs through MMP-2 [34]. MMP-2 also promoted the proliferation of HSCs through release of growth factors stored in the matrix [35]. Furthermore, MMP-2 has been shown to induce the release of VEGF [36], which is involved in hepatic sinusoidal capillarization and promotes the development of liver fibrosis [7,37–39]. Further study is important to investigate the mechanism underling the regulation of the expression and activity of MMP-2 in liver fibrosis.

## 5. Conclusions

In summary, in this study we provide several lines of evidence that ROS is an important factor released by hypoxic hepatocyte to regulate MMP-2 expression in HSCs, and NF-κB signaling is crucially involved in ROS-induced MMP-2 expression in HSCs. Our findings suggest that strategies aimed at antagonizing the generation of ROS in hypoxic hepatocytes and inhibiting NF-κB signaling in HSCs may represent novel therapeutic options for liver fibrosis.

## Figures and Tables

**Figure 7.**
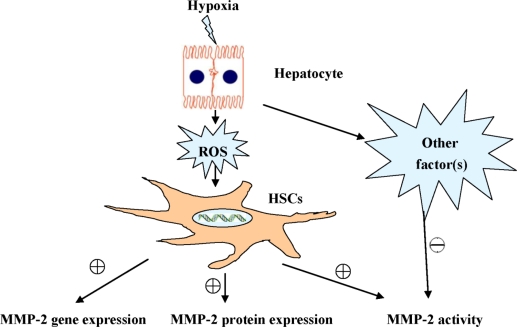
Hypoxic hepatocytes regulate MMP-2 expression and activity in Hepatic Stellate Cells.
